# What a first year for JCPP *Advances* – Time to celebrate!

**DOI:** 10.1002/jcv2.12085

**Published:** 2022-05-26

**Authors:** Henrik Larsson

**Affiliations:** ^1^ School of Medical Sciences Örebro University Örebro Sweden

Journal of Child Psychology and Psychiatry (JCPP) *Advances* is rapidly establishing itself as an international, high quality, high impact open access journal in the field of child psychology and psychiatry and related disciplines. We are making excellent progress towards our high ambitions, and what a first year we have had! It is time to celebrate. Since the launch of the first issue in April 2021 we have published over 70 articles including a mix of editorials, editorial perspectives, original articles, reviews and commentaries, with topics ranging from – the impact of school closures during Covid‐19, longitudinal studies on psychiatric referrals, to large population‐based cohort studies. Importantly, the published articles come from 13 countries, from several continents (Europe, North America, Australasia and Asia), helping our journal in the path of becoming truly international. I would like to take the opportunity to thank all of our authors, editors, reviewers, readers and the international research community who have supported us during this first year. I am really looking forward to continuing to work together during the coming years!

Our ambition to achieve our first Impact Factor remains an important goal, whilst embracing principles to encourage good authorship and highlight the individual contributions of a diverse range of researchers. We are committed to helping authors get published quickly and easily, which is also a reflection of the quality and impact of the journal. For example, successful authors who submit to JCPP *Advances* today will typically receive a first decision within 60 days. Our aim is that the work published in JCPP *Advances* will lead to a better understanding of, and better outcomes for, children, adolescents and young adults in our society. We aim to substantially increase the reach and impact of scientific advances. Our first year has been a great success in this regard, with researchers in over 160 countries accessing the journal, and downloads approaching 100,000. I am also pleased to share that every article published in JCPP *Advances* in 2021 generated wider impact via news articles, blogs and social media, as measured by Altmetric, and several publications have already found policy mentions making a difference in the real world. The most impactful article, as measured by Altmetric, published in JCPP *Advances* during 2021 showed that the COVID19 pandemic exacerbated mental health problems in already vulnerable children and that these negative outcomes were explained by financial stress which was negatively linked to parental mental health (Adegboye et al., 2021). As expected, several of the highest Altmetric scoring publications in JCPP *Advances* in 2021 had a similar goal; that is, to advance the understanding of how factors related to the COVID19 pandemic impacts on child and adolescent mental health. This topic will continue to be a critical research area also in the coming years.

JCPP *Advances* has in 2021 published important study findings from several internationally recognized cohort studies, such as the Avon Longitudinal Study of Parents and Children, Generation R, the Millennium Cohort Study and the Norwegian Mother, Father and Child Cohort Study (MoBa). The most‐read article in JCPP *Advances* during 2021 used MoBa data to explore the association between maternal acetaminophen use during and risk of ADHD in the child (Gustavson et al., 2021). A novel component of this study was the use of a sibling comparison design to examine the role of unmeasured familial confounding factors of this association. The study suggested that the observed association between long‐term acetaminophen use during pregnancy and ADHD in the child may at least partly be confounded by unobserved family factors (Gustavson et al., 2021). JCPP *Advances* will continue to showcase the very best research from studies using sophisticated statistical analyses of data from large‐scale prospective cohort studies combined with thoughtful study designs. Such studies plays a critical role in advancing our understanding about the causes, consequences and developmental trajectories of child and adolescent mental health problems.

JCPP *Advances* aims to lead and influence the field of child psychology and psychiatry and related disciplines. We work hard to identify trends and lead directions. We published our first special issue in December 2021 with a focus on the interplay between mental and physical health in children and adolescents. The main findings from the studies published in this first special issue were elegantly contextualized by an informative Editorial Perspective from Cortese et al. (2021) highlighting how multidisciplinary high‐quality work in this area could inform the integrated care for children and adolescents and elucidate underlying pathophysiological dimensions that are hoped to inform novel interventions to benefit the body and the mind in young people with mental and physical conditions.

Our second special issue, published in April 2022, showcased high‐quality research focused on sex and gender differences in neurodevelopmental and psychiatric phenotypes. The findings from the studies in this second special issue represent important building block to help improve clinical practice in terms of referral, timely diagnosis, and the development of effective prevention and intervention methods for the benefit of children and young people (Martin & Hadwin, 2022). For those with an interest in the topic I could highly recommend the accompanied editorial from Martin and Hadwin (2022), which not only provided a balanced context to the topic of this special issue, but also thoughtful directions for future research.

The third special issue of JCPP *Advances* is scheduled for late 2023 and will focus on high‐quality evidence‐synthesis studies of randomized clinical trials and observational data in child and adolescent mental health. Such evidence synthesis studies are critical for appraising available evidence and guiding clinical practice and policy around child and adolescent mental health problems. Another important goal of such evidence synthesis studies is to identify problems in primary research that should be addressed in future studies and also to provide directions for future research priorities. The publication of evidence‐synthesis studies has grown exponentially in recent decades, and many place such publications at the top of the pyramid of what is considered good evidence. Nevertheless, the quality of the published studies vary substantially and many are sensitive to bias and errors. The field therefore need careful discussions around key practical, conceptual and methodological issues related to conducting and reporting of high‐quality evidence‐synthesis studies.

There is no need to wait until late 2023 to access high‐quality evidence‐synthesis studies from JCPP *Advances*. We have during the last year published several informative narrative reviews and three high‐quality systematic reviews. For example, Senior et al. (2021) conducted a systematic review of new prediction models in child and adolescent mental health, and examined their development and validation (Senior et al., 2021), while Myers et al. (2021) evaluated the available evidence around mechanisms and phenotypic profiles associated with non‐shared environment effects in Autism spectrum disorder (Myers et al., 2021). Both studies were pre‐registered, carefully followed the Preferred Reporting Items for Systematic Reviews and Meta‐analyses, and included a risk of bias analysis, which are basic but important indicators of methodological quality. I happen to know that more high‐quality evidence‐synthesis studies will be published in JCPP *Advances* during 2022.

Authors publishing in JCPP *Advances* benefit from increased discoverability and accessibility of their research, by publishing in a Gold open access journal. All content published in JCPP *Advances* is freely available without charge to the user or their institution. We are committed to making research more open and efficient: users are allowed to read, download, copy, distribute, print, search, or link to the full texts of the articles, or use them for any other lawful purpose, without asking prior permission from the publisher or the author. This is in accordance with the BOAI definition of open access. We achieved our first milestone by being accepted for indexing in DOAJ (Directory of Open Access Journals) and we are now preparing applications for PMC in 2022 and MEDLINE in 2023. The journal has adopted many progressive initiatives like ‐ CRediT, mandating ORCiD, requiring data availability statements, offering open science badges and more. JCPP *Advances* is committed to scholarship that is respectful of diversity. The journal will strive to encourage wider representation on editorial board, editorial advisory board, reviewers and authors.

## CONFLICTS OF INTEREST

HL reports receiving grants from Shire Pharmaceuticals; personal fees from and serving as a speaker for Shire Pharmaceuticals and Evolan Pharma AB outside the submitted work; and sponsorship for a conference on attention‐deficit/hyperactivity disorder from Shire Pharmaceuticals outside the submitted work. He is also the Editor‐in‐Chief of JCPP *Advances*.
